# Maternal separation affects dopamine transporter function in the Spontaneously Hypertensive Rat: An in vivo electrochemical study

**DOI:** 10.1186/1744-9081-7-49

**Published:** 2011-12-01

**Authors:** Jacqueline S Womersley, Jennifer H Hsieh, Lauriston A Kellaway, Greg A Gerhardt, Vivienne A Russell

**Affiliations:** 1 Department of Human Biology, University of Cape Town, Observatory 7925 South Africa; 2 Department of Anatomy and Neurobiology, Center for Microelectrode Technology, Morris K. Udall Parkinson's Disease Research Center of Excellence, University of Kentucky Chandler Medical Center, Lexington, KY 40536-0098 USA

## Abstract

**Background:**

Attention-deficit/hyperactivity disorder (ADHD) is a developmental disorder characterised by symptoms of inattention, impulsivity and hyperactivity. The spontaneously hypertensive rat (SHR) is a well-characterised model of this disorder and has been shown to exhibit dopamine dysregulation, one of the hypothesised causes of ADHD. Since stress experienced in the early stages of life can have long-lasting effects on behaviour, it was considered that early life stress may alter development of the dopaminergic system and thereby contribute to the behavioural characteristics of SHR. It was hypothesized that maternal separation would alter dopamine regulation by the transporter (DAT) in ways that distinguish SHR from control rat strains.

**Methods:**

SHR and control Wistar-Kyoto (WKY) rats were subjected to maternal separation for 3 hours per day from postnatal day 2 to 14. Rats were tested for separation-induced anxiety-like behaviour followed by *in vivo *chronoamperometry to determine whether changes had occurred in striatal clearance of dopamine by DAT. The rate of disappearance of ejected dopamine was used as a measure of DAT function.

**Results:**

Consistent with a model for ADHD, SHR were more active than WKY in the open field. SHR entered the inner zone more frequently and covered a significantly greater distance than WKY. Maternal separation increased the time that WKY spent in the closed arms and latency to enter the open arms of the elevated plus maze, consistent with other rat strains. Of note is that, maternal separation failed to produce anxiety-like behaviour in SHR. Analysis of the chronoamperometric data revealed that there was no difference in DAT function in the striatum of non-separated SHR and WKY. Maternal separation decreased the rate of dopamine clearance (k_-1_) in SHR striatum. Consistent with this observation, the dopamine clearance time (T100) was increased in SHR. These results suggest that the chronic mild stress of maternal separation impaired the function of striatal DAT in SHR.

**Conclusions:**

The present findings suggest that maternal separation failed to alter the behaviour of SHR in the open field and elevated plus maze. However, maternal separation altered the dopaminergic system by decreasing surface expression of DAT and/or the affinity of DAT for dopamine, increasing the time to clear dopamine from the extracellular fluid in the striatum of SHR.

## Background

Attention-deficit hyperactivity disorder (ADHD) is a developmental disorder characterised by three predominant symptoms of hyperactivity, impulsivity and inattention that can manifest in behavioural problems later in life [1,2]. Though widely studied, the exact cause of ADHD is unknown. However, evidence from human studies and animal models suggests that dopamine is an important contributor to the aetiology of this disorder. Dopamine is associated with reward and in ADHD it is hypothesised that there is a disturbance in dopamine-mediated reinforcement of appropriate behaviour and extinction of unwanted behaviour manifesting in the behavioural symptoms of ADHD in certain families [3,4].

Genetic studies have focused chiefly on proteins involved in dopamine transmission [5], particularly genes that encode the dopamine transporter (DAT). Various polymorphisms in different regions of the DAT gene have been shown to be associated with increased risk of ADHD in some families [6,7]. DAT is responsible for clearing extracellular dopamine and therefore plays an important role in regulating those behaviours under the influence of the dopaminergic system.

The spontaneously hypertensive rat (SHR) is a well characterised genetic model of ADHD [8-11]. Consistent with the diagnostic criteria for ADHD, it displays hyperactivity, inattention and impulsivity [8,9]. Furthermore, it shares the dopamine hypofunction and subsequent altered behavioural reinforcement that is hypothesised to underlie the impulsivity and intolerance to delay seen in children with ADHD [3,9,12-15] The SHR was originally bred from the normotensive Wistar Kyoto (WKY) rat strain, which provides a suitable control for the SHR [10,16,17]. The WKY strain used in this study was obtained from Harlan, UK, and has been recommended as the most appropriate reference strain for SHR [17].

Similar to patients with ADHD who displayed a variable number of tandem repeats in the 3'-untranslated region of the DAT gene [6,18-21], SHR have been shown to have a 160 bp insertion in the non-coding region of the DAT gene, upstream of exon 3, suggesting altered regulation of transcription of the DAT gene [22]. Consistent with this observation is the finding that DAT expression is transiently reduced in SHR midbrain during the first postnatal month and increased in adult SHR relative to controls [23,24]. It has however not been shown whether increased DAT expression alters dopamine clearance. It was therefore of interest to measure DAT function in vivo using chronoamperometric recording of the clearance of a known amount of pressure ejected dopamine in SHR and WKY striatum [25-27].

Since ADHD is considered to result from a complex interaction between small gene effects and the environment [28] rats were subjected to stress during the early stages of development, to determine whether early life trauma would alter their behaviour and DAT function [29]. Maternal separation is a well-developed model of early life trauma, simulating the effects of adverse early life experiences on behaviour and neurobiology [29,30]. Separation of pups from the dam during the early postnatal period interrupts postnatal care at a stage when pups are dependent on the dam for warmth and nutrition. Coupled with continuing development of the brain and dopamine neurons, this represents a period of high vulnerability [31-34]. One of the major consequences of maternal separation is increased depression- and anxiety-like behaviour in adulthood [35-38], which is likely to involve changes in the limbic system, including the mesolimbic dopaminergic system.

Maternal separation has been shown to have opposite effects on behaviour of SHR and WKY in the elevated plus maze [39]. Maternal separation increased the activity of SHR in the novel environment of the maze, while it decreased that of WKY. The aim of the present study was to test the hypothesis that early life stress alters behaviour and DAT regulation of dopamine function differently in SHR and WKY. DAT function was measured in maternally separated and non-separated SHR and WKY striatum, using in vivo electrochemical recording of the disappearance of a known amount of dopamine.

## Methods

### Materials

Ascorbic acid, dopamine-HCl, urethane and Nafion^® ^were purchased from Sigma Chemical Company (St. Louis, MO, U.S.A). All other chemicals were of the highest standard and were purchased from Merck Chemicals (Germany). Carbon fibre electrodes were purchased from Quanteon LLC (Lexington, KY, U.S.A). Teflon-coated silver wire was purchased from A-M Systems (Carlsborg, WA, U.S.A).

### Animals

Three month old male and female inbred WKY (Harlan, UK) and SHR rats (Charles River Laboratories, USA) were obtained from the University of Cape Town Animal Unit and paired in cages for breeding. The date of birth of the litter was designated postnatal day 0 (P0). On P2, litters were culled to 8 pups to ensure equal nourishment during the early postnatal period. Male rats were preferentially selected to avoid the confounding effects of fluctuating hormones during the female oestrus cycle on brain function and behaviour in later experiments. This selection produced an average of 4.3 males and 3.7 females in WKY NMS litters, 4.6 males and 3 females in WKY MS litters, 6.3 males and 1.2 females in SHR NMS litters, and 5.2 males and 1.7 females in SHR MS litters. On P2, litters were designated as either maternally separated experimental rats or non-maternally separated control rats to give a total of 21 non-separated WKY; 22 maternally separated WKY; 20 non-separated SHR and 29 separated SHR. Rats were housed in the University of Cape Town satellite animal facility in cages with woodchip bedding and a 12 hour light/dark cycle (lights on at 06h00). Rats had *ad libitum *access to food and water. This study was conducted in accordance with international guidelines (South African National Standard: The care and use of animals for scientific purposes. 1^st ^edition, 2008) and approved by the University of Cape Town Faculty of Health Sciences Animal Ethics Committee.

### Maternal separation

The maternal separation protocol involved removal of the dam from the pups for 3 hours per day between 09h00 and 13h00 from P2 to P14 [40]. Pups were transferred in the home cage to a different room to prohibit communication with the dam by means of ultrasound vocalisation [40]. The temperature within the home cage was maintained at 31-33°C by infrared heating lamps so as to avoid the risk of possible hypothermia. After 3 hours the pups were returned to the animal facility and the dams returned to their home cages. Rats designated as controls were maintained in the home cage at all times until weaning. The cleaning routine was standard across both separated and non-separated rats with approximately half of the soiled wood-chip bedding removed every third day in the first week and every second day in the second and third weeks following birth. Thus handling of pups was consistent across both maternally separated and non-separated experimental groups. On P21 rats were weaned and male rats separated from their female littermates (the ratio of male to female pups in maternally separated and non-separated litters did not differ significantly within strains but SHR litters tended to have a higher ratio of male to female pups than WKY). The male rats were housed in groups of two to four rats per cage for the remainder of the project.

### Behavioural testing

On P28, rats were taken to the behavioural facility and allowed to acclimatise to the behavioural testing room for a minimum of 1 hour prior to testing in the open field and elevated plus maze.

The open field test measures total distance covered in a 100 cm × 100 cm (floor) × 50 cm (walls) black box, as well as time spent and number of entries into the inner zone (70 cm × 70 cm) of the box. The tests were conducted in an isolated noise-free room. The lighting in the room was 50 lux. More time spent in the inner zone indicated increased exploration, and was defined as reduced anxiety [41,42]. The distance covered provided an indication of the rat's locomotor activity [41,42]. Each rat was tested in a single trial of 5 minutes. All behavioural tests were recorded with a Sony Handicam DCR-SX 83E for later analysis with Noldus Ethovision XT 7.0 (Noldus Information Technology, Wageningen, The Netherlands).

After the open field test, the rat was returned to the home cage and a minimum of 1 hour was allowed to pass before the rat was tested in the elevated the plus maze which has open and closed arms, elevated 50 cm above the ground. The animal was placed in the centre of the maze, facing an open arm and allowed to explore the maze for 5 minutes. Time spent in the open arms suggested decreased anxiety-like behaviour while time spent in the closed arms suggested increased anxiety-like behaviour [43]. Gloves were used throughout the behavioural experiments. The maze was cleaned after every test with 70% alcohol to ensure that the rat's behaviour was not affected by the scent of another rat.

### In vivo Electrochemical Measurement of Dopamine Clearance

High-speed in vivo chronoamperometric measurement of extracellular dopamine was carried out using the FAST-16 system (Quanteon LLC, Lexington, KY, U.S.A) with Nafion-coated carbon fibre microelectrodes (electrode tips, 30 μm outer diameter and 150 μm length) [44,45]. This technique has the benefit of high spatial resolution and second-by-second temporal resolution, which make it superior to in vivo microdialysis coupled with high-performance liquid chromatography techniques [46]. The Nafion^® ^coating (5% solution in alcohol at 200°C) was used to enhance the sensitivity and selectivity of the electrodes for dopamine over other electroactive molecules [45,47]. Prior to use in vivo, electrodes were calibrated in 0.05 M phosphate-buffered saline solution (10 mM NaH_2_PO_4_, 40 mM Na_2_HPO_4_, 100 mM NaCl) to which a known amount of ascorbic acid and incremental amounts of dopamine were added to determine selectivity of the electrodes for dopamine and to generate a calibration curve of current versus dopamine concentration.

A square wave potential (0 to +0.55 V vs. a glass RE-5 Ag/AgCl reference electrode) was applied to the carbon fibre microelectrode for 100 ms to create a 5 Hz waveform repeated at 1 Hz intervals to cause the oxidation and subsequent reduction of dopamine at the microelectrode surface. Dopamine has a specific red/ox ratio (~0.7-0.9) which was used as a 'chemical fingerprint' to confirm specific dopamine recording [47]. Changes in extracellular concentrations of dopamine in vivo were expressed as changes from a stable baseline response of the microelectrode. Only carbon fibre microelectrodes that gave a selectivity for dopamine over ascorbic acid greater than 250:1, a limit of detection less than 0.1 μM dopamine, and a correlation coefficient greater than 0.997, were used (Table 1). A glass micropipette (1 mm od, 0.58 mm id) was pulled and bumped to produce a micropipette tip with an inner diameter of approximately 10 μm. The microelectrode and micropipette were aligned in parallel and joined by sticky wax (Kerr Corporation, Orange, CA, USA) such that their tips were 180 - 220 μm apart. A miniature Ag/AgCl reference electrode was prepared from Teflon-coated silver wire by removing ~3 mm of the Teflon sheath at one end, exposing the tip and anodizing it at +10 volts versus platinum wire in a solution of 1 M HCl saturated with NaCl for 10-15 minutes. The anodized Ag/AgCl reference electrodes were stored in 3 M NaCl prior to use in vivo [48].

**Table 1:** Carbon fibre electrode calibration parameters

	Median	Lower quartile	Upper quartile
Selectivity for dopamine over ascorbic acid	622:1	356:1	781:1

Limit of detection (μM)	0.0190	0.0076	0.0300

Correlation coefficient	0.9995	0.9993	0.9997

Calibration parameters are displayed as median, 25% and 75% quartiles.

### Measurement of Dopamine Uptake in SHR and WKY striatum

Between P49 and P54 rats were deeply anesthetized with 25% urethane at a dose of 1.25 g/kg to produce non-recoverable terminal anaesthesia. Urethane was chosen because it affected the activity of neurotransmitter systems less than other anaesthetic agents [49]. Rats were placed in a stereotactic frame, on a heated cushion maintained at 37°C by circulating water. A midline incision was made, the scalp was reflected and a burr hole drilled to provide access to the right striatum. A burr hole was similarly drilled above the left posterior cortex for the placement of a miniature Ag/AgCl reference electrode. The microelectrode assembly was positioned at +1 mm anterior to Bregma, 2.5 mm lateral to the sagittal suture and lowered 3 mm into the rat brain under stereotactic guidance using the flat skull coordinates of Paxinos and Watson [50]. High-speed chronoamperometric electrochemical measurements were continuously made with the carbon fibre microelectrode using a FAST16 mkI recording system (Quanteon, LLC, Lexington, Kentucky, USA). After a baseline period of 1 hour, a 200 μM dopamine solution (0.2 mM dopamine plus 100 μM ascorbic acid, in normal saline adjusted to pH 7.2-7.4) was ejected into the striatum using a Picospritzer II (Parker Instrumentation, Parker Hannifin Corporation). The volume of ejected dopamine was varied by finely adjusting the pressure and time controls on the picospritzer to achieve a peak amplitude between 0.75 and 1.5 μM dopamine. The microelectrode assembly was lowered by 0.5 mm increments from 3.5 to 5.0 mm ventral to the cortical surface and striatal clearance of a pressure ejected pulse of dopamine was recorded.

### Data Analysis

**Figure 1:**
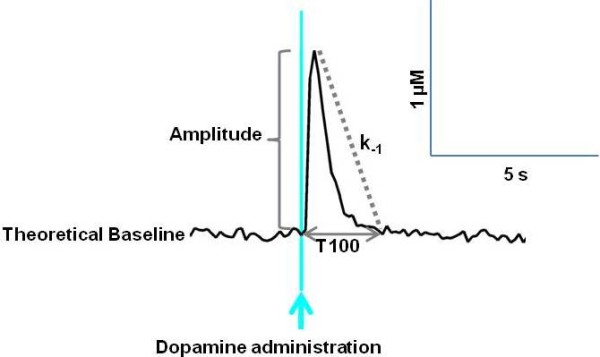
**Representative graph showing dopamine clearance in rat striatum**. The amplitude of the peak was measured as the maximum change in dopamine concentration from baseline. T100 represents the time taken for the dopamine concentration to return from maximum amplitude to baseline. The k_-1 _is the first order rate constant. It provides a measure of the rate of decay of dopamine concentration over time, to provide a measure of DAT efficiency.

The amplitude, defined as the difference between baseline and peak dopamine concentration, first-order rate constant (k_-1_), and clearance time (T100) were measured (Figure 1). The first-order rate constant k_-1 _is calculated from the decay of dopamine concentration versus time. It is indicative of dopamine uptake efficiency and as such, is indirectly proportional to the clearance time [51,52]. T100 represents the time taken for the dopamine concentration to return from peak amplitude to the baseline value prior to the ejection of dopamine. Independent recordings of dopamine clearance were made at 0.5 mm intervals (between 3.5 and 5 mm ventral to the cortical surface) in the striatum. A total of 38 recordings were obtained from 10 non-separated WKY (WKY NMS), 24 recordings from 7 maternally separated WKY (WKY MS), 25 recordings from 7 non-separated SHR (SHR NMS) and 31 recordings from 8 maternally separated SHR (SHR MS).

After completion of the experiment, the rats were cervically dislocated and decapitated. The brain was removed from the skull and placement of the electrodes confirmed to be in the rat striatum and thus all data resulting from these recordings were included in the analysis.

### Statistics

All data were analysed using Statistica 10. The Shapiro-Wilk's test was used to test for normality and all data were found to be non-normally distributed. Therefore non-parametric statistics in the form of the Kruskal-Wallis and Mann-Whitney U tests were used for between group comparisons. Significance was defined as p < 0.05. Due to the data being non-normally distributed, results are displayed as median and interquartile range. Graphs were prepared using Graph Pad Prism 5.

## Results

### Behaviour

#### Open field test

**Figure 2:**
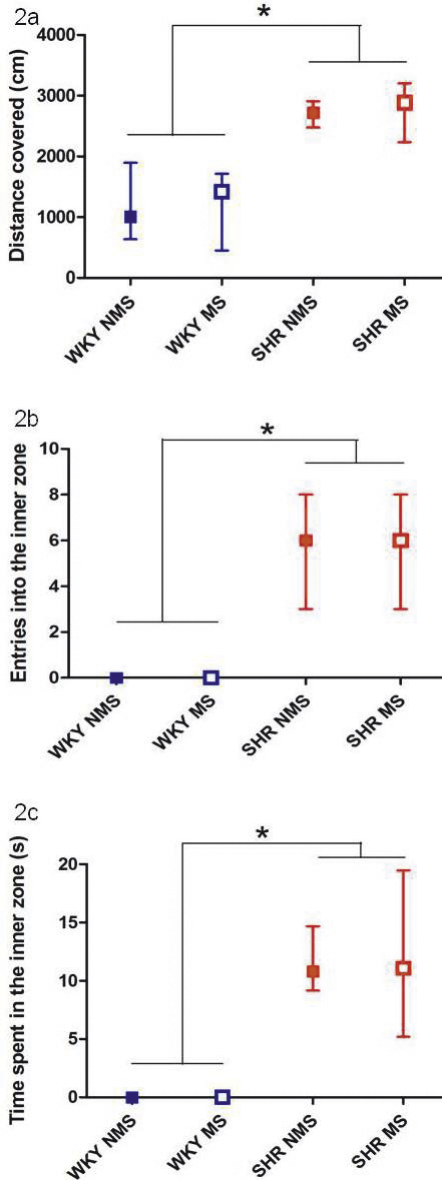
**The SHR were more active than WKY in the open field**. Results are displayed as the median and interquartile range. a. Total distance covered during the 5 minutes of the open field test grouped by strain and stress. *SHR covered a significantly greater distance than WKY rats (p < 0.0001). b. Number of entries into the inner zone of the open field during the 5 minutes of testing grouped by strain and stress. *SHR entered the inner zone more frequently than WKY rats (p < 0.0001). c. Time spent in the inner zone of the open field during the 5 minutes of testing. *SHR spent significantly more time in the inner zone than WKY (p < 0.001).

SHR covered a significantly greater distance than WKY in the open field (Kruskal-Wallis H (3, N = 92) = 50.1, p < 0.0001, Mann-Whitney U test, p < 0.0001, Figure 2a). This difference between strains was consistent over the 5 minute test period.

The total number of entries into the inner zone of the open field over the 5 minute test period was significantly greater for SHR compared to WKY rats (Kruskal-Wallis H (3, N = 92) = 64.5, p < 0.0001, Mann-Whitney U test, p < 0.0001, Figure 2b). SHR also spent more time in the inner zone of the open field than WKY rats (Kruskal-Wallis H (3, N = 92) = 59.7, p < 0.0001, Mann-Whitney U test, p < 0.001, Figure 2c).

#### Elevated plus maze

**Figure 3:**
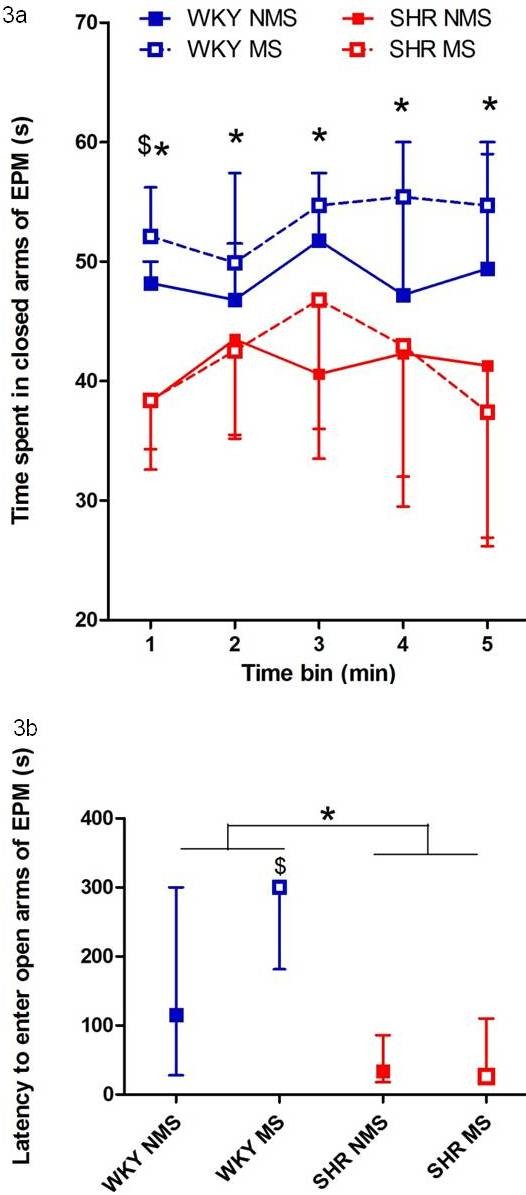
**Maternally separated WKY rats showed increased anxiety-like behaviour in the elevated plus maze relative to non-separated WKY controls**. Results are displayed as median and interquartile range. a. Time spent in the closed arms of the elevated plus maze grouped by strain and stress and divided into 5 × 1 minute time bins. * SHR spent significantly less time in the closed arms than WKY in all time periods (p < 0.05). **^$^**Maternally separated WKY rats spent significantly more time in the closed arms than non-separated WKY in the first minute (p < 0.05). b. Latency to enter the open arms of the elevated plus maze grouped by strain and stress. *SHR took a significantly shorter time to enter the open arm than WKY rats (p < 0.0001). **^$^**Maternally separated WKY rats took significantly longer to enter the open arm of the elevated plus maze than WKY NMS rats (p < 0.05).

SHR spent less time in the closed arms of the elevated plus maze than WKY (Kruskal-Wallis H (3, N = 92) = 21.1, p < 0.001, Mann-Whitney U test, p < 0.0001, Figure 3a). When the test was analysed in 1 minute time bins, maternally separated WKY rats were seen to spend more time in the closed arms in the first minute than non-maternally separated WKY (Kruskal-Wallis H (3, N = 92) = 24.6, p < 0.0001, Mann-Whitney U test, p < 0.05, Figure 3a).

SHR displayed a shorter latency to enter the open arms of the elevated plus maze than WKY (Kruskal-Wallis H (3, N = 92) = 27.6, p < 0.0001, Mann-Whitney U test, p < 0.0001, Figure 3b). Maternally separated WKY rats took significantly longer to enter the open arms of the elevated plus maze than non-maternally separated WKY rats (Mann-Whitney U test, p < 0.05, Figure 3b).

### Dopamine Uptake Studies using Chronoamperometry

#### Striatum (3.5 mm to 5 mm DV)

**Figure 4:**
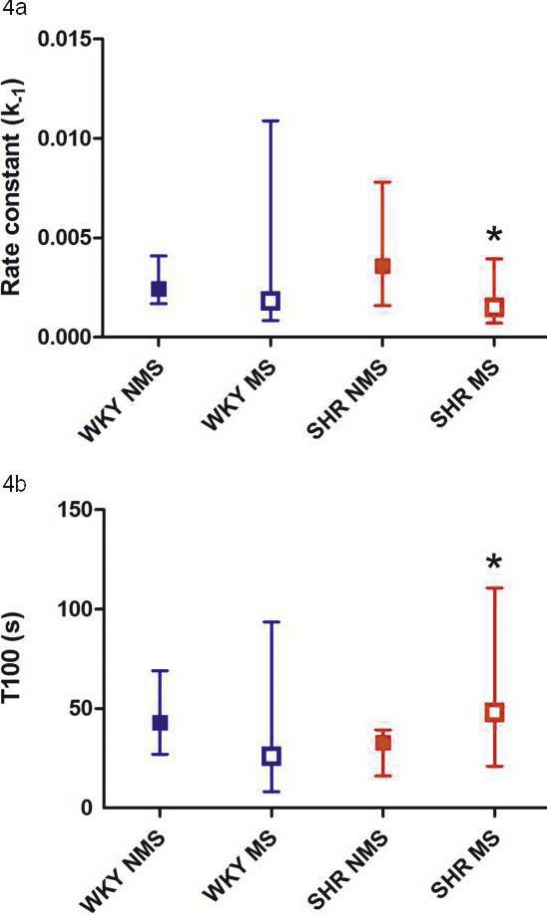
**Maternal separation reduced the rate of dopamine uptake in SHR striatum**. Results are displayed as median and interquartile range. a. The rate constant (k**_-1_**) for dopamine clearance (DAT function) in SHR and WKY striatum (at depths of 3.5 to 5 mm DV) was decreased by maternal separation in SHR. *MS significantly lower than NMS (p < 0.05). b. Total clearance time (T100) for exogenous dopamine ejected into the striatum (3.5 to 5 mm DV) in maternally separated and non-separated SHR and WKY. *Maternal separation increased T100 in SHR compared to non-separated SHR (p < 0.05).

The rate of dopamine clearance by DAT, k_-1_, was similar in SHR and WKY striatum (Kruskal-Wallis H (1, N = 118) = 1.3, p = 0.262) (Table 2). Maternal separation did not alter the rate of clearance of dopamine (Kruskal-Wallis H (3, N = 118) = 7.0, p = 0.072). However, when SHR striatal data were analysed separately, a significantly lower rate constant for dopamine clearance (k_-1_) was observed in maternally separated SHR compared to non-separated SHR (Mann-Whitney U test, p < 0.05, Figure 4a). In support of this finding, the time required for the dopamine concentration to return to baseline (T100) was increased in maternally separated SHR compared to non-maternally separated SHR (Mann-Whitney U test, p < 0.05, Figure 4b). Thus, maternal separation appeared to decrease the surface expression and/or function of DAT in SHR striatum.

**Table 2:** Dopamine clearance parameters in rat striatum

	Amplitude (μM)	k_-1_			T rise (s)			T50 (s)			T80 (s)			T100 (s)		
	**Mean ± SEM**	**Median**	**Lower quartile**	**Upper quartile**	**Median**	**Lower quartile**	**Upper quartile**	**Median**	**Lower quartile**	**Upper quartile**	**Median**	**Lower quartile**	**Upper quartile**	**Median**	**Lower quartile**	**Upper quartile**

WKY NMS	1.1187 ± 0.0320	0.0025	0.0017	0.0041	3.00	2.00	4.00	6.00	4.00	9.00	9.00	7.00	16.00	43.00	27.00	69.00

WKY MS	1.1777 ± 0.0342	0.0018	0.0008	0.0109	3.00	2.00	7.00	4.50	3.00	10.67	8.50	4.00	23.17	26.00	8.17	93.50

SHR NMS	1.1653 ± 0.0469	0.0036	0.0016	0.0078	2.00	2.00	4.67	6.00	3.33	7.33	11.00	7.00	16.00	33.00	16.00	39.33

SHR MS	1.1407 **± **0.0354	0.0015	0.0007	0.0039	3.00	2.00	6.00	7.00	4.00	15.00	16.00	6.00	40.00	48.00	21.00	110.66

* Amplitudes of dopamine peaks were normally distributed, while k_-1_, T rise, T50, T80 and T100 data were non-normally distributed (Shapiro-Wilks test). k_-1 _is the first-order rate constant of dopamine decay versus time. T rise represents the time taken for the dopamine concentration to reach the peak value. T50 represents the time taken for the dopamine concentration to return from peak amplitude to 50% of peak value. T80 represents the time taken for the dopamine concentration to return from peak amplitude to 20% of peak value. T100 represents the time taken for the dopamine concentration to return from peak amplitude to the baseline value prior to the ejection of dopamine

## Discussion

Consistent with the behavioural characteristics of ADHD and in agreement with previous studies, SHR displayed increased exploratory activity and decreased measures of anxiety in the open field and elevated plus maze compared to WKY rats [16,39]. In agreement with previous findings, SHR did not develop anxiety-like behaviour in response to the chronic mild stress of maternal separation [39]. Anxiety could be masked by their impulsivity or increased activity in a novel environment, as previously suggested [39]. In contrast, maternally separated WKY rats took longer to enter the open arms of the elevated plus maze and spent more time in the closed arms of the maze in the first minute of testing in comparison to non-separated WKY, similar to the effects of maternal separation on other rat strains [39,53,54]

Maternal separation decreased the rate of clearance of exogenously applied dopamine in SHR striatum. The k_-1 _was decreased and it took longer to clear a known amount of dopamine in SHR striatum, suggesting that the mild chronic developmental stress of maternal separation had decreased DAT efficiency and/or reduced surface expression of DAT. Postnatal development of dopamine terminals in the striatum, and hence DAT surface expression, occurs rapidly between P7 and P35 with smaller subsequent increases in DAT to reach adult levels at P60 [55]. The timing of maternal separation overlaps the period of rapid development of the dopaminergic system and may therefore alter DAT expression. SHR have been suggested to have impaired dopamine function, yet there was no difference between non-separated SHR and WKY in the rate of clearance of exogenously applied dopamine, suggesting that DAT function is unimpaired in non-separated SHR striatum at P50. This may be due to increased expression of DAT in SHR striatum which is impeded by the early life trauma of maternal separation. Strain differences in the rate of development of the dopaminergic system have been found with tyrosine hydroxylase mRNA reduced in the midbrain of the SHR in the first postnatal week of development in comparison to WKY control rats [23] and increased in adulthood [24].

These findings suggest that SHR have impaired DAT function, which is not apparent under normal conditions but becomes apparent when the individual is subjected to early developmental stress in the form of maternal separation. Environmental factors can therefore influence development of the dopaminergic system giving rise to impaired DAT function and possibly lead to ADHD-like symptoms. In fact, patients with ADHD have been shown to have decreased striatal DAT availability compared to controls [56]. Furthermore, disruption of the dopamine reward pathway has been associated with motivation deficits in adults with ADHD and suggested to contribute to their difficulty in sustaining attention [57].

## Conclusions

SHR displayed increased behavioural activation relative to WKY, consistent with SHR being a widely accepted model of ADHD. WKY displayed increased anxiety-like behaviour, consistent with the anxiogenic effects of maternal separation previously observed in WKY and other rat strains. Of note is that SHR did not display effects of maternal separation in behavioural tests of anxiety suggesting that SHR were resilient to the anxiogenic effects of chronic mild developmental stress. In contrast, maternal separation decreased the rate of clearance of dopamine in SHR striatum. SHR appear to have impaired DAT function, which is not apparent under normal conditions but becomes apparent when the individual is subjected to early developmental stress in the form of maternal separation.

## Competing interests

The authors have no competing interests except for the fact that GAG owns Quanteon, LLC, makers of the FAST recording system that was used to carry out measures of dopamine uptake.

## Authors' contributions

JSW contributed to the design of the study, carried out experimental procedures, analysed data and drafted the manuscript. JHH assisted with the experimental procedures and execution of the study. GAG, LAK and VAR contributed to the development, design and execution of the study, and analysis of the data. All authors have contributed to the drafting of the manuscript and have read and approved the final manuscript.
